# Expedited transfer pathways vs. standard care for adults with out-of-hospital cardiac arrest: a systematic review and meta-analysis of randomized trials

**DOI:** 10.3389/fmed.2026.1917259

**Published:** 2026-08-14

**Authors:** Anzhen Wang, Li Chen, Xihan Li

**Affiliations:** 1 College of Health, Medicine and Wellbeing, The University of Newcastle, Newcastle, NSW, Australia; 2 The Third School of Clinical Medicine, Nanjing Medical University, Nanjing, China

**Keywords:** expedited transfer, extracorporeal cardiopulmonary resuscitation, meta-analysis, out-of-hospital cardiac arrest, transportation of patients

## Abstract

**Background:**

In adults with out-of-hospital cardiac arrest (OHCA), the clinical benefit of expedited transfer compared with standard resuscitation pathways remains uncertain. This study aimed to evaluate randomized controlled trial evidence on the effects of expedited transfer on survival and neurological outcomes.

**Methods:**

PubMed, MEDLINE, Embase, and Scopus were searched from inception to 22 February 2026. Eligible studies were randomized controlled trials comparing expedited transfer with standard care in adults with prehospital OHCA. The primary outcome was all-cause mortality at the longest reported follow-up; secondary outcomes included neurological recovery, bleeding, mechanical circulatory support, time to return of spontaneous circulation, and length of hospital stay. Random-effects meta-analyses were performed; risk of bias was assessed using RoB 2, certainty of evidence using GRADE. Subgroup analyses were conducted according to clinical pathway type, comparing post-ROSC transfer to cardiac arrest centers with intra-arrest transport pathways for refractory OHCA.

**Results:**

Four randomized controlled trials involving 1,303 patients were included. Compared with standard care, expedited transfer did not significantly reduce all-cause mortality at the longest reported follow-up (RR = 0.97, 95% CI: 0.90–1.04) or improve favorable neurological outcome at hospital discharge (RR = 0.98, 95% CI: 0.81–1.18). No statistically significant between-group differences were observed for the remaining secondary outcomes. Sensitivity analyses were consistent with the primary analysis. Subgroup analysis showed no significant interaction according to expedited transfer pathway type (P for subgroup difference = 0.46).

**Conclusion:**

Current randomized evidence does not support routine expedited transfer as a standard strategy for adults with OHCA.

## 1 Introduction

Out-of-hospital cardiac arrest (OHCA) remains a major global public health challenge, with persistently high mortality and low overall survival (1). Despite ongoing advances in cardiopulmonary resuscitation (CPR) techniques and advanced life support (ALS) strategies, a substantial proportion of patients do not achieve return of spontaneous circulation (ROSC) during the initial prehospital resuscitation phase, including those with refractory OHCA and some patients with OHCA without ST-segment elevation (2, 3). These patients generally have a poor prognosis, and outcomes are strongly influenced by the duration of the low-flow state, defined as the period during which organ perfusion remains critically inadequate despite ongoing CPR (4). Increasing evidence suggests that prolonged low-flow duration is closely associated with irreversible neurological injury and multiorgan dysfunction (5, 6).

Against this background, expedited transfer (including intra-arrest transport) has emerged as a potential strategy to improve outcomes in this patient population (7). This approach emphasizes early transfer of patients to specialized cardiac arrest centers (CACs) while maintaining high-quality CPR, often with the assistance of mechanical chest compression devices (8). At such centers, patients may gain earlier access to advanced interventions, including coronary angiography (CAG), percutaneous coronary intervention (PCI), and extracorporeal cardiopulmonary resuscitation (ECPR), thereby increasing the opportunity to identify and treat reversible causes in a timely manner (9).

However, the clinical effectiveness of expedited transfer remains uncertain, and the available evidence has not reached a consistent conclusion (10). Several observational studies have suggested that early transfer of patients with OHCA to centers capable of advanced resuscitative care may confer a survival advantage (8). In contrast, more recent randomized controlled trials (RCTs), including INCEPTION (11) and PRAGUE-OHCA (12), have not demonstrated a significant advantage of expedited transfer over continued on-scene resuscitation in terms of survival or neurological outcomes (13). Differences in emergency medical services (EMS) organization, patient selection, prehospital resuscitation pathways, and the potential deterioration in CPR quality during transport may all contribute to these inconsistent findings and to heterogeneity across studies (10).

Accordingly, a systematic synthesis of the currently available randomized evidence is needed to re-evaluate the effectiveness of expedited transfer. In this systematic review and meta-analysis, we compared expedited transfer with continued on-scene resuscitation or standard care in patients with refractory OHCA or OHCA without ST-segment elevation, with a particular focus on survival and neurological outcomes. We also explored whether the treatment effect differed between post-ROSC transfer pathways and intra-arrest transport pathways.

## 2 Methods

### 2.1 Protocol and reporting

This systematic review and meta-analysis was conducted in accordance with the Preferred Reporting Items for Systematic Reviews and Meta-Analyses (PRISMA) statement (14). The protocol was prospectively registered in the International Prospective Register of Systematic Reviews (PROSPERO) (registration number: CRD420261323805).

### 2.2 Search strategy and information sources

We systematically searched PubMed, MEDLINE, Embase, and Scopus from inception to 22 February 2026. The search strategy combined controlled vocabulary terms, including Medical Subject Headings (MeSH) where applicable, with free-text terms related to out-of-hospital cardiac arrest, prehospital care, patient transport, intra-arrest or expedited transfer, and randomized controlled trials. Online-ahead-of-print articles were eligible. No restrictions were applied by language, country, or region.

To identify additional studies, we manually screened the reference lists of included articles and relevant systematic reviews. No additional eligible randomized controlled trials were identified through manual screening of the reference lists of the included studies and relevant systematic reviews. Two reviewers (XL and LC) independently screened records in duplicate. Disagreements were resolved by discussion and, when required, by consultation with a third reviewer (AW).

### 2.3 Eligibility criteria

Population: adults (≥18 years) with out-of-hospital cardiac arrest (OHCA);

Intervention: expedited transfer pathways, including direct post-ROSC transfer to a cardiac arrest center or intra-arrest transport with mechanical CPR to centers capable of invasive assessment, coronary angiography, percutaneous coronary intervention (PCI), extracorporeal cardiopulmonary resuscitation (ECPR), or mechanical circulatory support;

Comparator: standard local emergency medical services (EMS) pathways, including transfer to the nearest emergency department, extended on-scene advanced life support (ALS), or transport according to usual care criteria;

Outcomes: the primary outcome was all-cause mortality at the longest reported follow-up. Secondary outcomes included favorable neurological outcome (e.g., cerebral performance category 1–2), bleeding, length of hospital stay, time from arrest to return of spontaneous circulation (ROSC), use of mechanical circulatory support, and other reported mortality outcomes at available follow-up time points;

Study design: randomized controlled trials (RCTs) only. Ongoing trials without available outcome data and conference abstracts without sufficient methodological and outcome information for eligibility assessment and data extraction were excluded.

### 2.4 Study selection

After duplicate removal, two reviewers (XL and LC) independently screened titles and abstracts, followed by full-text assessment of potentially eligible reports. Eligibility was determined against prespecified inclusion and exclusion criteria. Disagreements were resolved by discussion; unresolved discrepancies were adjudicated by a third reviewer (AW).

### 2.5 Data extraction

Two reviewers (XL and LC) independently extracted data using a prespecified standardized form. Extracted variables included study characteristics (first author, year, country, design), sample size, group allocation, baseline characteristics, details of intervention and comparator pathways, and prespecified outcome data. Baseline variables included age, sex, bystander cardiopulmonary resuscitation (CPR), and mechanical CPR, where available.

For continuous outcomes reported as medians with interquartile ranges or ranges, means and standard deviations were estimated using established methods when appropriate (15, 16). Extracted data were cross-checked by a third reviewer (AW), and discrepancies were resolved by consensus.

### 2.6 Risk of bias assessment

Risk of bias was assessed independently by two reviewers (XL and LC) using the Cochrane Risk of Bias 2 (17) tool. The following domains were evaluated: bias arising from the randomization process; bias due to deviations from intended interventions; bias due to missing outcome data; bias in outcome measurement; and bias in selection of the reported result.

Overall judgments followed the RoB 2 algorithm and were classified as low risk of bias, some concerns, or high risk of bias. Disagreements were resolved by discussion, with arbitration by a third reviewer (AW) where required.

### 2.7 Certainty of evidence

Certainty of evidence for each outcome was assessed independently by two reviewers (XL and LC) using the Grading of Recommendations Assessment, Development and Evaluation (18) framework. Certainty was rated as high, moderate, low, or very low across the domains of risk of bias, inconsistency, indirectness, imprecision, and publication bias. Disagreements were resolved by discussion, with third-reviewer adjudication (AW) when necessary.

### 2.8 Statistical analysis

All analyses were performed using Review Manager (RevMan), version 5.4.2 (Cochrane Collaboration, London, United Kingdom), in accordance with the prespecified review protocol. Given anticipated clinical and methodological heterogeneity across trials, random-effects models were used throughout.

For dichotomous outcomes, pooled estimates were expressed as risk ratios (RRs) with 95% confidence intervals (CIs). For continuous outcomes, pooled estimates were expressed as mean differences (MDs) with 95% CIs. Statistical significance was defined as a two-sided *p* < 0.05.

Between-study heterogeneity was quantified using the I^2^ statistic and interpreted as low (<25%), moderate (25–50%), substantial (50–75%), or considerable (>75%) (19, 20). Because fewer than 10 studies were available, funnel plots and formal tests for small-study effects were not performed (21). For the primary outcome of all-cause mortality, the longest available follow-up reported by each trial was used for the main synthesis. Mortality outcomes at specific follow-up time points were analyzed separately where sufficient data were available.

### 2.9 Subgroup analyses

We performed an exploratory subgroup analysis for the primary outcome according to the type of expedited transfer strategy. Trials were grouped as either post-ROSC transfer to a cardiac arrest center for non-ST-elevation OHCA, including Patterson et al. (22) and Patterson et al. (23), or intra-arrest transport for refractory OHCA during ongoing resuscitation, including Belohlavek et al. (2) and Burns et al. (24). Subgroup effects were compared using the test for subgroup differences in Review Manager. Because only two trials were available in each subgroup, these analyses were considered exploratory.

### 2.10 Sensitivity analyses

Three prespecified sensitivity analyses were performed:

(1) replacement of the random-effects model with a fixed-effect model;
(2) restriction to studies at low risk of bias after exclusion of studies rated as some concerns or high risk of bias;
(3) leave-one-out analysis, in which each study was sequentially omitted to examine the influence of individual trials on the pooled estimate and on the direction and statistical significance of the overall result.

## 3 Results

### 3.1 Study selection and characteristics

**Figure 1:**
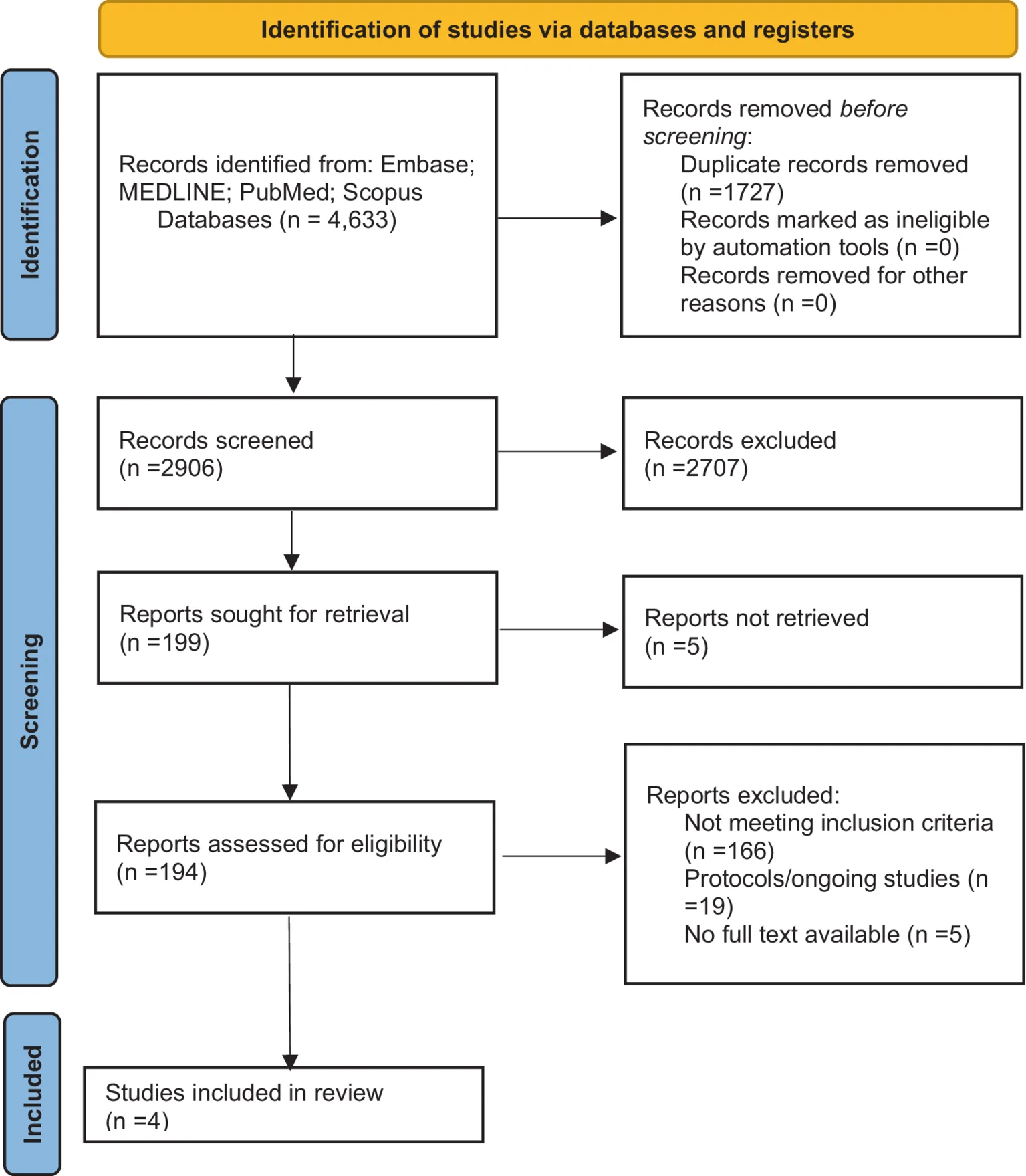
Flow diagram of study selection.

The initial literature search identified 4,633 records. After removal of duplicates, 2,906 records remained for screening. Following title, abstract, and full-text assessment, four randomized controlled trials (RCTs) met the eligibility criteria and were included in the systematic review and meta-analysis (Figure 1).

The main characteristics of the included trials are summarized in Table 1. The four RCTs were published between 2017 and 2025 and were conducted in the United Kingdom, the Czech Republic, and Australia (2, 22–24). Sample sizes ranged from 33 to 823 participants.

**Table 1:** Baseline characteristics of the included randomized controlled trials comparing expedited transfer versus standard care in out-of-hospital cardiac arrest.

Authors	Year	Design	Country	Sample size	Age (year)	Female (%)	Bystander CPR	Mechanical CPR	Population	Intervention	Comparison	Outcomes
Patterson T, et al.	2017	RCT	UK	33	61 ± 15	5 (14%)	22 (61%)	17 (47%)	Adults with witnessed shockable (VT/VF) OHCA.	Expedited transfer to a Cardiac Arrest Centre (CAC) for immediate reperfusion.	Standard transport strategy	30-day all-cause mortality and trial feasibility.
Belohlavek J, et al.	2022	RCT	Czech Republic	256	57 ± 13.3	44 (17%)	252 (98.4%)	218 (85.2%)	Adults (18–65 years) with witnessed OHCA without ROSC	Intra-arrest transport, ECPR, and immediate invasive treatment.	Standard transport strategy	180-day survival with good neurologic outcome
Patterson T, et al.	2023	RCT	UK	823	63.5 **±** 16	262 (32%)	603 (72.9%)	193 (23.4%)	Adults (≥18 years) with ROSC after OHCA, without ST elevation.	Expedited transfer to a CAC	Standard transport strategy	30-day all-cause mortality.
Burns B, et al.	2025	RCT	Australia	197	56 ± 12.6	36 (18%)	195 (99%)	194 (98.5%)	Adults with refractory OHCA (initial shockable rhythm or PEA)	Expedited intra-arrest transport to a cardiac catheterization center	Standard transport strategy	Survival with good neurological outcome (CPC 1–2) at hospital discharge

Clinical and methodological heterogeneity was present across the included studies. Mean participant age ranged from 56 to 63.5 years, and the proportion of women ranged from 14 to 32%. Rates of bystander cardiopulmonary resuscitation (CPR) varied from 61 to 99%, and use of mechanical CPR ranged from 23.4 to 98.5%. Definitions and assessment time points for neurological outcomes also differed across trials (2, 22–24). Although all studies used the cerebral performance category (CPC) scale and generally defined favorable neurological outcome as CPC 1–2, one study (23) additionally reported the modified Rankin Scale (mRS), while another incorporated longer-term quality-of-life and cognitive assessments (24). All four studies (2, 22–24) reported all-cause mortality at the longest reported follow-up, three (22–24) reported favorable neurological outcome at discharge and 30-day neurological outcome, two (2, 24) reported 6-month neurological outcome, and three (2, 22, 24) reported bleeding events.

### 3.2 Primary outcomes

**Figure 2:**
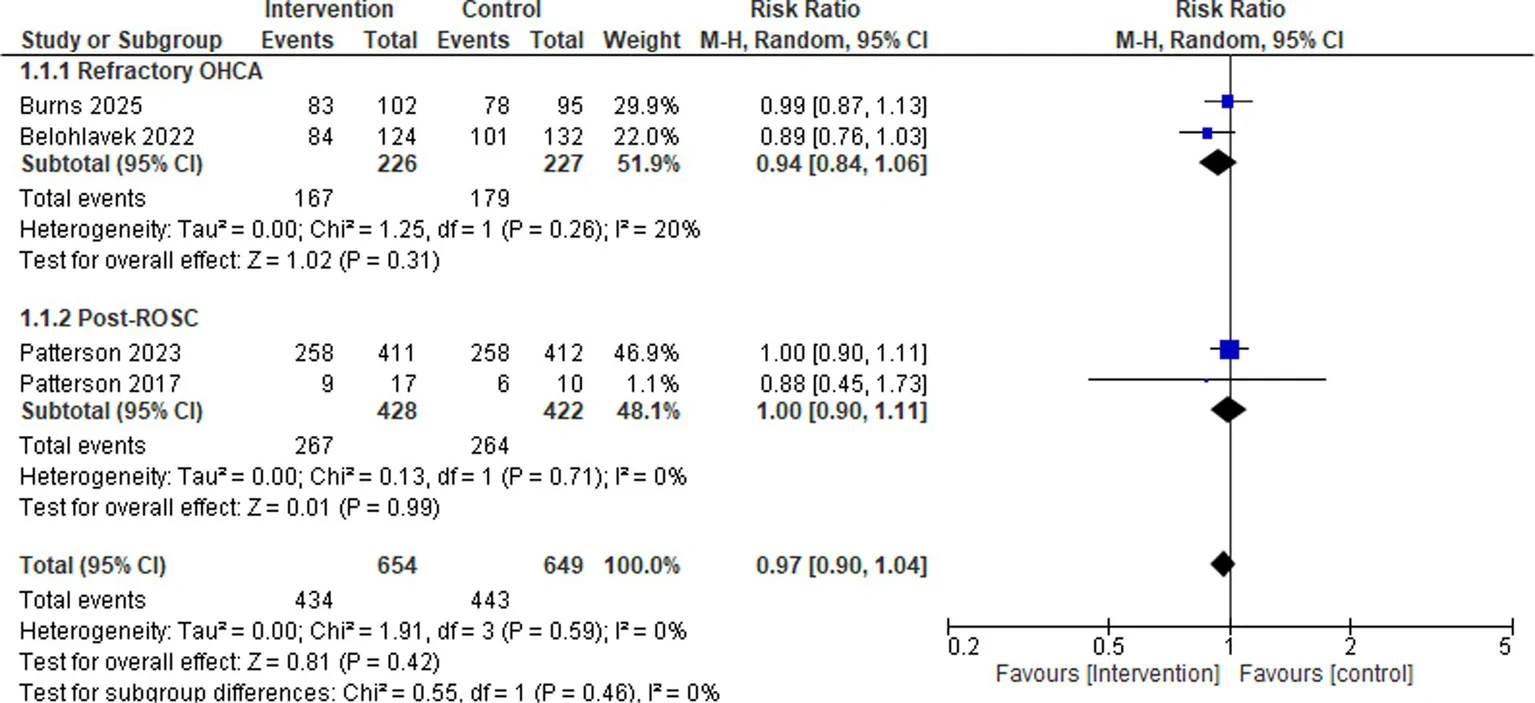
Forest plot of subgroup analysis for all-cause mortality at the longest reported follow-up, stratified by clinical pathway (refractory OHCA versus post-ROSC).

A total of 1,303 patients from four trials (2, 22–24) were included in the analysis of the primary outcome of all-cause mortality at the longest reported follow-up. Of these, 654 patients were allocated to the expedited transfer group and 649 to the standard care group. Death occurred in 434/654 patients (66.4%) in the expedited transfer group and 443/649 (68.3%) in the standard care group. Pooled analysis showed that expedited transfer was not associated with a statistically significant reduction in all-cause mortality at the longest reported follow-up compared with standard care (RR 0.97, 95% CI 0.90–1.04) (Figure 2).

**Figure 3:**
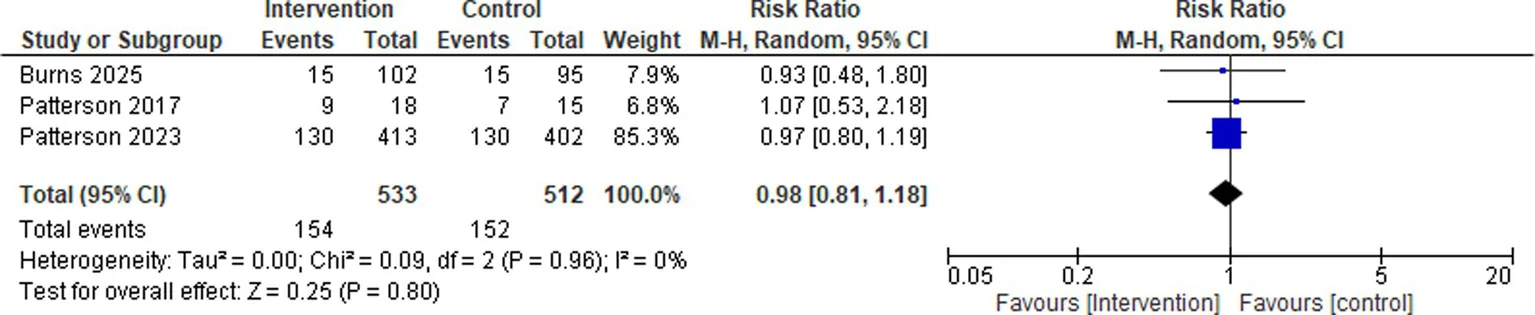
Forest plot comparing favorable neurological outcome at hospital discharge between expedited transfer and standard care in adults with out-of-hospital cardiac arrest.

Regarding favorable neurological outcome at hospital discharge, data from three studies (22–24) involving 1,045 patients were available. In the expedited transfer group, 154/533 patients (28.9%) achieved favorable neurological outcomes, compared with 152/512 (29.7%) in the standard care group. There was no significant difference between groups (RR 0.98, 95% CI 0.81–1.18) (Figure 3).

### 3.3 Subgroup analyses

Subgroup analyses were performed to determine whether the effect of expedited transfer on all-cause mortality at the longest reported follow-up differed according to the clinical pathway evaluated.

In the refractory OHCA subgroup, which included studies evaluating intra-arrest transport during ongoing resuscitation, expedited transfer was not associated with a statistically significant reduction in mortality compared with standard care (RR 0.94, 95% CI 0.84–1.06; *I*^2^ = 20%) (Figure 2). Similarly, in the post-ROSC subgroup, which included studies evaluating transfer to a cardiac arrest center after return of spontaneous circulation, expedited transfer did not reduce mortality compared with standard care (RR 1.00, 95% CI 0.90–1.11; *I*^2^ = 0%) (Figure 2).

The formal test for subgroup differences demonstrated no significant interaction between the two clinical pathway categories (P for subgroup difference = 0.46), indicating that the overall effect of expedited transfer did not differ significantly between post-ROSC transfer strategies and intra-arrest transport strategies.

Although the pooled effect estimates differed numerically between subgroups, the confidence intervals overlapped substantially and no evidence was found to support a differential treatment effect according to expedited transfer pathway type.

### 3.4 Secondary outcomes

**Figure 4:**
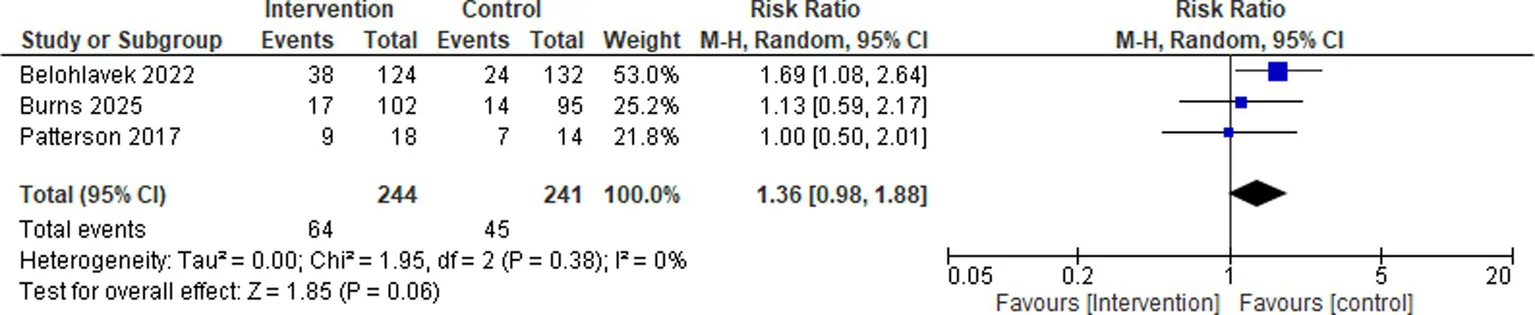
Forest plot comparing 30-day favorable neurological outcome (CPC 1–2) between expedited transfer and standard care in adults with out-of-hospital cardiac arrest.

**Figure 5:**
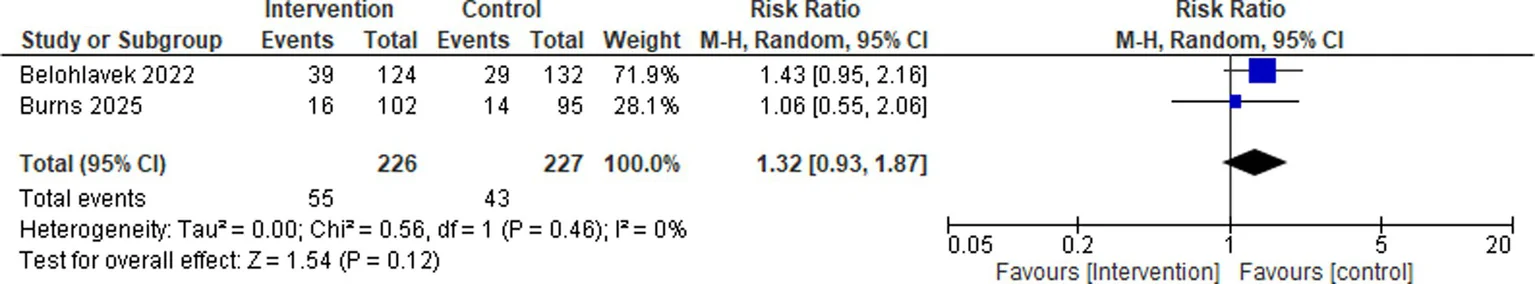
Forest plot comparing 6-month favorable neurological outcome (CPC 1–2) between expedited transfer and standard care in adults with out-of-hospital cardiac arrest.

**Figure 6:**
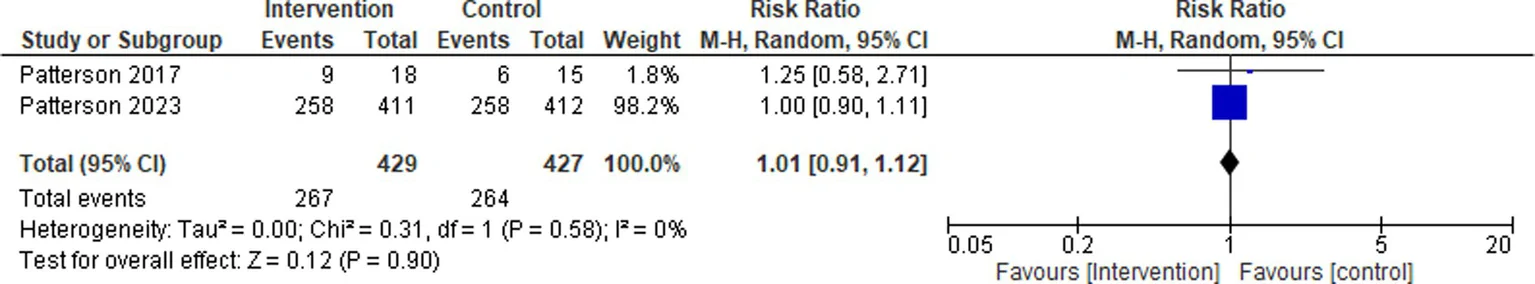
Forest plot comparing 30-day all-cause mortality between expedited transfer and standard care in adults with out-of-hospital cardiac arrest.

**Figure 7:**
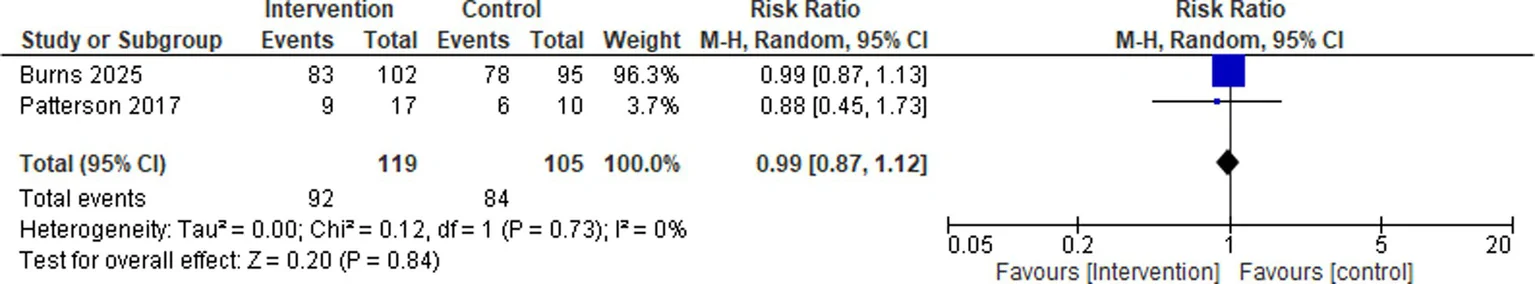
Forest plot comparing 6-month all-cause mortality between expedited transfer and standard care in adults with out-of-hospital cardiac arrest.

No significant between-group differences were observed across the secondary outcomes assessed. For neurological outcomes, expedited transfer was not associated with improved 30-day favorable neurological outcome (RR 1.36, 95% CI 0.98–1.88) (Figure 4) or 6-month favorable neurological outcome (RR 1.32, 95% CI 0.93–1.87) (Figure 5). Similarly, no significant differences were observed for 30-day all-cause mortality (RR 1.01, 95% CI 0.91–1.12) (Figure 6) or 6-month all-cause mortality (RR 0.99, 95% CI 0.87–1.12) (Figure 7).

**Figure 8:**
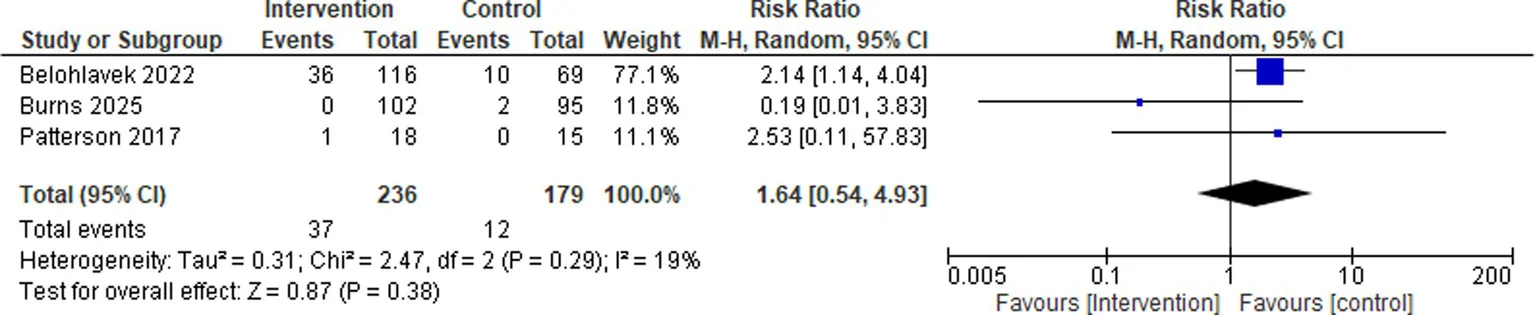
Forest plot comparing bleeding events between expedited transfer and standard care in adults with out-of-hospital cardiac arrest.

**Figure 9:**
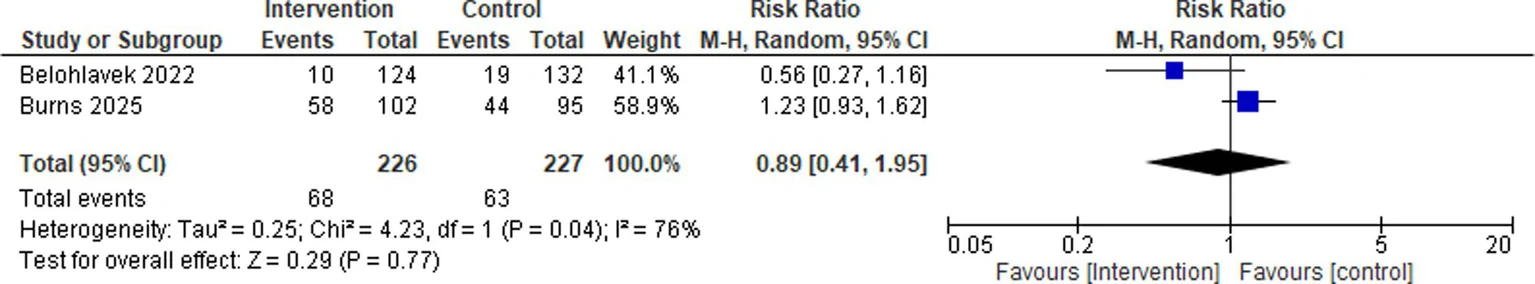
Forest plot comparing death within 1 h of hospital arrival between expedited transfer and standard care in adults with out-of-hospital cardiac arrest.

**Figure 10:**
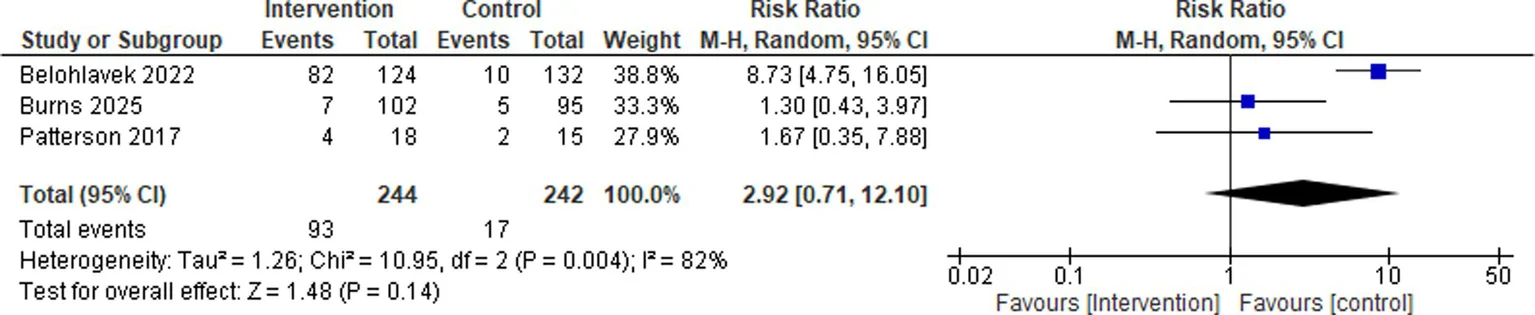
Forest plot comparing mechanical circulatory support use between expedited transfer and standard care in adults with out-of-hospital cardiac arrest.

**Figure 11:**
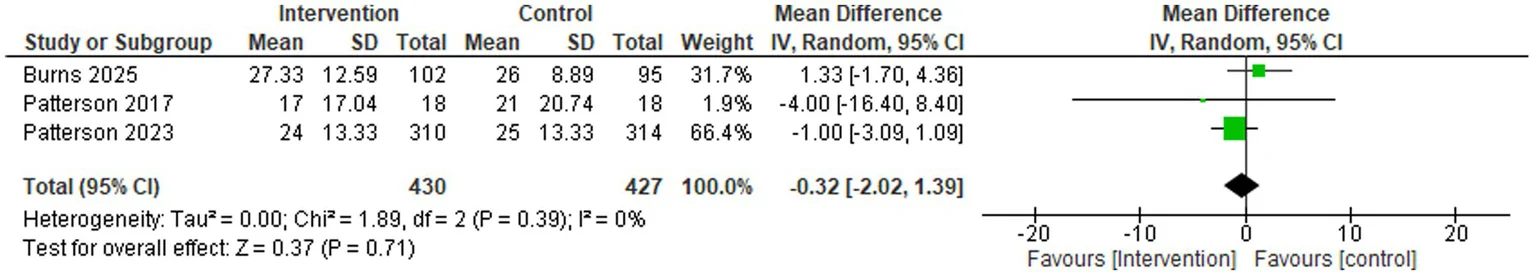
Forest plot comparing time from cardiac arrest to return of spontaneous circulation (ROSC) between expedited transfer and standard care in adults with out-of-hospital cardiac arrest.

**Figure 12:**
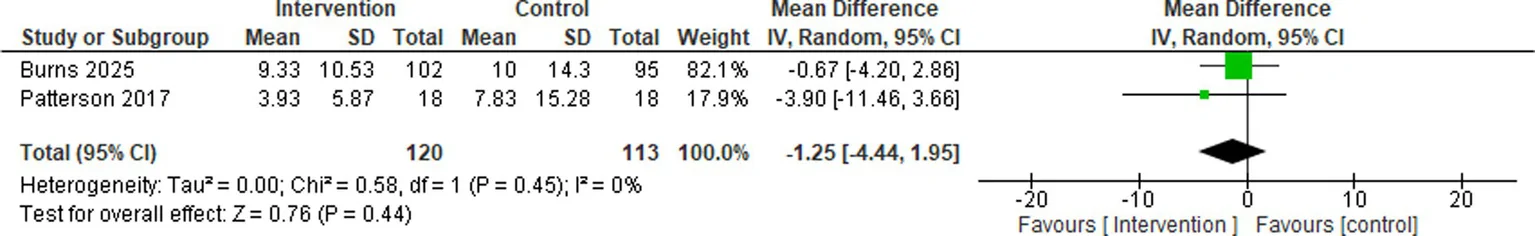
Forest plot comparing length of hospital stay between expedited transfer and standard care in adults with out-of-hospital cardiac arrest.

With respect to safety and resource-related outcomes, expedited transfer was not associated with significant differences in bleeding events (RR 1.64, 95% CI 0.54–4.93) (Figure 8), death within 1 h of hospital arrival (RR 0.89, 95% CI 0.41–1.95) (Figure 9), mechanical circulatory support use (RR 2.92, 95% CI 0.71–12.1) (Figure 10), time from cardiac arrest to ROSC (MD −0.32, 95% CI −2.02 to 1.39) (Figure 11), or length of hospital stay (MD −1.25, 95% CI −4.44 to 1.95) (Figure 12).

### 3.5 Sensitivity analyses

Sensitivity analyses yielded findings consistent with the primary analysis. Replacement of the random-effects model with a fixed-effect model produced a similar pooled estimate for all-cause mortality at the longest reported follow-up (RR 0.97, 95% CI 0.90–1.05) (Table 2). The influence of the small ARREST pilot trial was examined in the leave-one-out sensitivity analysis, and its exclusion did not materially alter the direction or magnitude of the pooled estimate. Restricting the analysis to studies judged to be at low risk of bias also yielded consistent findings (RR = 1.00, 95% CI: 0.92–1.08) (Table 2).

**Table 2:** Sensitivity analysis of meta-analysis.

	No. patients (trials)	RR	95%CI
All trials	1,303 (4)	0.97	0.90, 1.04
Using fixed-effect models	1,303 (4)	0.97	0.90, 1.05
Excluding studies with high risk of bias or some concerns.	1,020 (2)	1.00	0.92, 1.08
Excluding each randomized control trial in turn			
Excluding Patterson et al. (22)	1,276 (3)	0.97	0.90, 1.04
Excluding Belohlavek et al. (2)	1,047 (3)	1.00	0.92, 1.08
Excluding Patterson et al. (23)	480 (3)	0.94	0.85, 1.04
Excluding Burns et al. (24)	1,106 (3)	0.96	0.88, 1.05

### 3.6 Risk of bias and certainty of evidence

**Figure 13:**
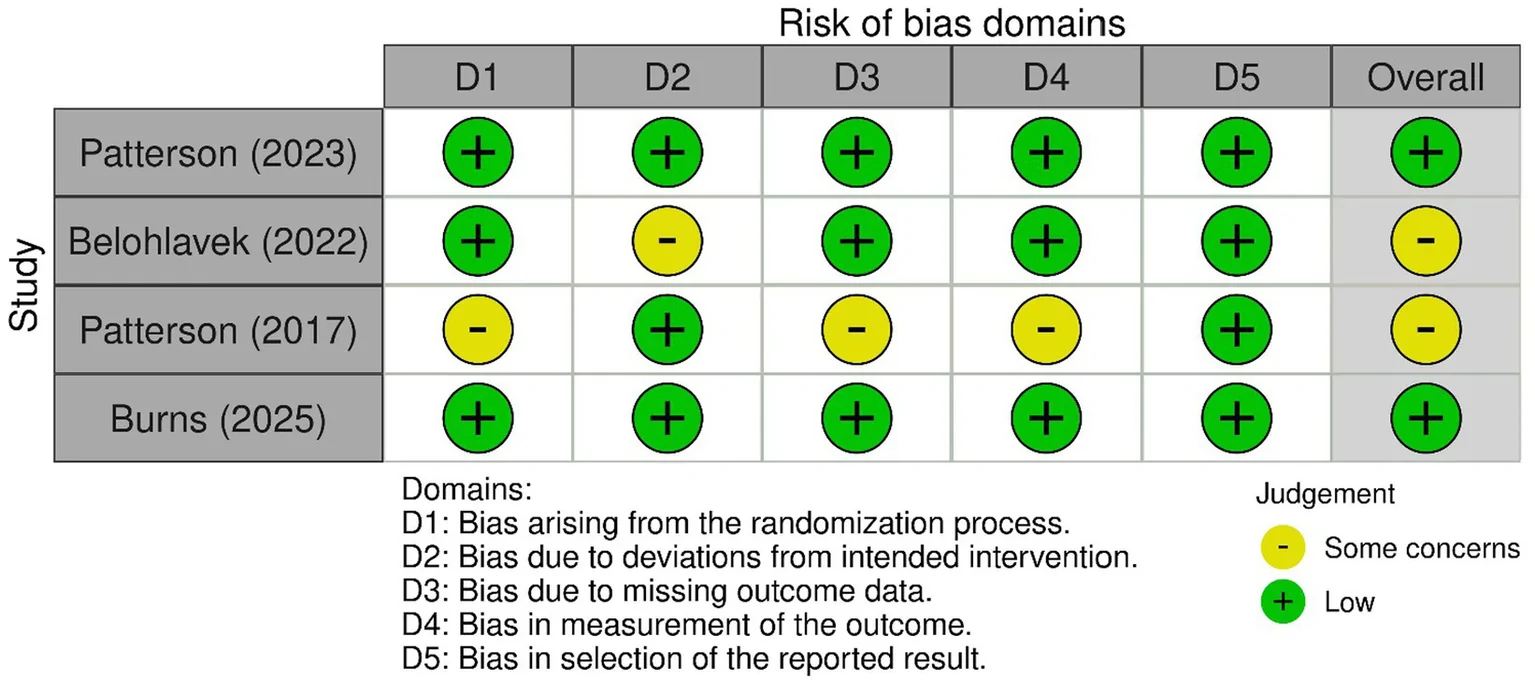
Risk of bias summary.

Risk-of-bias assessments are summarized in Figure 13. Of the four included RCTs (2, 22–24), two (23, 24) were judged to be at low risk of bias and two (2, 22) were judged as raising some concerns. No study was judged to be at high risk of bias.

According to the GRADE framework, the certainty of evidence was rated as high for 30-day all-cause mortality and 6-month all-cause mortality, and moderate for all-cause mortality at the longest reported follow-up. The certainty of evidence for the primary outcome was downgraded by one level for indirectness because the included trials evaluated clinically distinct populations and heterogeneous transfer pathways across different EMS and hospital systems, and mortality was assessed at different follow-up time points. The certainty of evidence was rated as low-to-moderate for favorable neurological outcome at discharge, low for bleeding events, CPC 1–2 at 30 days, CPC 1–2 at 6 months, and declaration of death within 1 h of hospital arrival, and very low for mechanical circulatory support (Table 3).

**Table 3:** Summary of findings and strength of evidence.

Outcome	No. of patients (trials)	Relative effect, risk ratio (95%MD/CI)	Absolute effect estimates (per 1,000)			Quality of the evidence
			Intervention	Control	Difference	
The primary outcome						
All-cause mortality at the longest reported follow-up	1,303(4)	0.97(0.9, 1.04)	664	683	−20 [−68, 27]	Moderate*
Good neurological function at discharge	1,045(3)	0.98(0.81, 1.18)	289	297	−6[−56,53]	Moderate*
The secondary outcome						
Bleeding	415(3)	1.64(0.54, 4.93)	157	67	43 [−31,263]	Low*
CPC 1–2 at 30 days	485(3)	1.36(0.98, 1.88)	262	187	67[−4,164]	Low*
All-cause mortality at 30 days	856(2)	1.01(0.91, 1.12)	622	618	6[−56,74]	High*
CPC 1–2 at 6 months	453(2)	1.32(0.93, 1.87)	243	189	61[−13,165]	Low*
All-cause mortality at 6 months	224(2)	0.99(0.87, 1.12)	773	800	−8[−104,96]	High*
Declaration of death within 1 h of hospital arrival	453(2)	0.89(0.41, 1.95)	301	278	−31[−164,264]	Low*
Mechanical circulatory support	486(3)	2.92(0.71, 12.1)	381	70	135[−20,780]	Very low*
Time from arrest to ROSC	857(3)	−0.32(−2.02, 1.39)				
Length of stay	233(2)	−1.25(−4.44, 1.95)				

*Downgraded one level for indirectness because the included trials evaluated clinically distinct pathways, including post-ROSC transfer to a cardiac arrest center and intra-arrest transport for refractory OHCA.

## 4 Discussion

In this systematic review and meta-analysis of four randomized trials (2, 22–24) involving 1,303 patients with OHCA, expedited transfer was not associated with improved survival or favorable neurological outcomes compared with standard care. Specifically, no significant benefit was observed for the primary outcome of all-cause mortality at the longest reported follow-up or for favorable neurological outcome at hospital discharge. Across secondary outcomes, including short-term and longer-term mortality, neurological recovery, bleeding, mechanical circulatory support, and time-to-ROSC measures, no statistically significant differences were identified.

These overall findings should be interpreted in the context of two clinically distinct transport phenotypes included in the expedited-transfer strategy: patients with refractory OHCA transported during ongoing cardiopulmonary resuscitation and patients transferred after achieving ROSC without ST-segment elevation. These populations differ substantially in physiological state, treatment objectives, and logistical requirements. Intra-arrest transport occurs during an ongoing low-flow state and remains dependent on continuous, high-quality chest compressions (2). Patient loading, transfer into the ambulance, ambulance movement, equipment constraints, and interruptions associated with transport may compromise CPR quality even when mechanical chest compression devices are used (5, 6). These transport-related challenges could therefore disproportionately attenuate the potential benefit of expedited transfer in the intra-arrest phenotype (6). In contrast, post-ROSC transport occurs after restoration of spontaneous circulation and is not dependent on uninterrupted external chest compressions during ambulance movement, although these patients may remain clinically unstable. Although the formal subgroup interaction test did not demonstrate a statistically significant difference between the two phenotypes (*p* = 0.46), only two trials contributed to each subgroup, resulting in limited statistical power. The absence of a statistically significant interaction should therefore not be interpreted as evidence of equivalent treatment effects. Accordingly, the pooled estimate represents an average effect across heterogeneous intra-arrest and post-ROSC pathways and should not be interpreted as evidence that expedited transfer provides no benefit in every OHCA phenotype.

This distinction is clinically important because the biological rationale for expedited transfer remains compelling (25). Early transfer to a specialized cardiac arrest center could, in principle, shorten the time to definitive treatment by facilitating access to coronary angiography, percutaneous coronary intervention, extracorporeal cardiopulmonary resuscitation, and other advanced postarrest interventions (2, 23, 26). However, the present RCT-based evidence suggests that these potential advantages do not consistently translate into improved patient-centered outcomes across the heterogeneous populations and transfer pathways evaluated.

Our findings differ from signals reported in earlier observational studies suggesting benefit from early transfer to specialized centers (8). A likely explanation is that observational comparisons in this field are highly susceptible to selection bias, resuscitation-time bias, and confounding by differences in prehospital triage, transport feasibility, and receiving-center capability (23). By restricting the present meta-analysis to randomized trials, we aimed to provide a more internally valid estimate of the effect of expedited transfer. The absence of a clear benefit across randomized evidence therefore strengthens the inference that expedited transfer should not currently be viewed as a universally superior strategy.

Several explanations may account for the lack of observed benefit. Expedited transfer is a complex system-level intervention rather than transport alone, and its effectiveness depends on maintaining high-quality CPR, limiting transport duration, early cardiac arrest center activation, adherence to the allocated pathway, timely cannulation, and access to coronary intervention or ECPR. Evidence regarding bystander CPR emphasizes the importance of early, simple, and minimally interrupted chest compressions (27); however, this pre-transport phase should be distinguished from the subsequent loading and transport phase, during which compression quality may deteriorate despite mechanical devices. Initial rhythm may also modify the balance between transport and continued on-scene resuscitation, as a recent rhythm-stratified observational study suggested different associations in shockable and nonshockable OHCA (28). Variation in CPR quality, transport duration, center activation, treatment crossover, cannulation delay, and access to definitive intervention may therefore attenuate the treatment contrast. Consequently, a neutral intention-to-treat estimate may reflect either a limited overall effect of the expedited-transfer strategy itself or dilution of a potentially beneficial effect because of incomplete pathway implementation or system-related delays. First, transport during ongoing resuscitation may compromise the continuity or quality of CPR despite the use of mechanical chest compression devices (25). Second, earlier arrival at a cardiac arrest center does not necessarily guarantee timely delivery of advanced interventions within the narrow therapeutic window needed to influence outcomes (23).

Importantly, the null pooled effect should not be interpreted as a direct estimate of the efficacy of ECPR, because the included trials evaluated heterogeneous expedited-transfer pathways, and ECPR was only one component of the bundled intervention in the refractory-OHCA trials. Within ECPR-enabled systems, patient selection and system performance are likely to be interdependent. Potential benefit may depend on initial rhythm, witnessed status, bystander CPR, no-flow and anticipated low-flow durations, the presence of a reversible cardiac cause, and ECPR eligibility. Patients who might otherwise benefit may lose that opportunity because of delays in recognition, transport, center activation, or cannulation, whereas even a highly efficient pathway may not improve outcomes if eligibility criteria do not adequately identify patients with reversible pathology and a favorable no-flow and anticipated low-flow profile (29, 30). Therefore, neutral findings in broadly selected populations do not exclude benefit in carefully selected patients, particularly those with shockable rhythms and short resuscitation intervals. Given the small number and heterogeneity of the included trials and the use of aggregate data, the present analysis cannot determine the relative contributions of system-level barriers and patient selection to the null pooled effect. These findings are broadly consistent with current ILCOR, AHA, and ERC guidance, which supports cardiac arrest center transfer according to local protocols and limits consideration of rapid intra-arrest transport to carefully selected ECPR candidates within organized regional systems, rather than recommending routine expedited transfer for all patients with refractory OHCA (31, 32).

The secondary outcome findings further support a cautious interpretation. Although point estimates for some neurological outcomes numerically favored expedited transfer, confidence intervals crossed unity and no statistically significant differences were demonstrated. Similarly, no clear safety signal was identified for bleeding, and no consistent effect was observed for resource-intensive outcomes such as mechanical circulatory support. Heterogeneity was greater for outcomes such as early in-hospital death and mechanical circulatory support use, which may reflect variation in local postarrest management pathways, thresholds for advanced support, and termination-of-resuscitation practices across studies. These outcomes should therefore be interpreted with caution.

The present study has several strengths. Unlike the 2023 systematic review by Burns et al. (10), which relied largely on observational studies, the present review incorporates more recent randomized evidence and is restricted to RCTs, thereby reducing susceptibility to confounding from observational evidence. We also performed prespecified sensitivity analyses, and the main findings remained stable across fixed-effect modelling, leave-one-out analyses, and restriction to lower-risk studies.

This study also has limitations. First, the included trials differed in patient populations and intervention pathways; some focused on refractory OHCA, whereas others enrolled patients with OHCA without ST-segment elevation, and the content of the expedited transfer pathway varied across systems. Second, definitions and follow-up time points for important outcomes were not fully uniform across studies, which limits direct comparability. Third, only four randomized trials were available, and several secondary outcomes were informed by only two or three studies, resulting in residual imprecision. In addition, individual participant-level data were unavailable, precluding examination of treatment effects according to initial rhythm, witnessed status, bystander CPR, no-flow or low-flow duration, the presence of a potentially reversible cause, or eligibility for ECPR. Although publication bias was not formally assessed because fewer than 10 studies were available, it cannot be excluded. Selective non-publication of small or inconclusive trials may have affected the magnitude or direction of the pooled estimates. Fourth, because the included interventions represented complex care pathways, blinding of prehospital and hospital clinicians was not feasible. Knowledge of treatment allocation may therefore have influenced co-interventions and downstream management, including activation of cardiac arrest centers, access to coronary angiography or ECPR, use of mechanical circulatory support, and decisions regarding withdrawal of life-sustaining treatment. These effects may have been more relevant to secondary outcomes and exploratory subgroup findings than to the primary mortality outcome. Finally, all included RCTs were conducted in high-resource settings, which may limit generalizability to health systems with different EMS structures or limited access to specialized cardiac arrest centers.

Taken together, the present evidence does not support the routine adoption of expedited transfer as a universally superior alternative to continued on-scene resuscitation or standard care pathways in adults with OHCA. Any potential benefit may be restricted to specific settings or carefully selected patient subgroups, rather than applying broadly across all patients with OHCA. Further large, methodologically consistent, multicenter randomized trials are needed to clarify whether particular phenotypes of OHCA or particular system configurations may derive benefit from this strategy.

## 5 Conclusion

Based on currently available randomized evidence, expedited transfer was not associated with a clear survival or neurological benefit in adults with OHCA. These findings do not support routine use of expedited transfer as a universally superior resuscitation strategy over standard care. Future research should focus on identifying the patient subgroups, transport conditions, and system-level characteristics most likely to influence the effectiveness of this approach.
